# Transforming neonatal care: a position paper on the potential of augmented and mixed reality

**DOI:** 10.3389/fdgth.2025.1571521

**Published:** 2025-04-11

**Authors:** Patrik Goncalves Rodrigues, Danieli Mayumi Kimura Leandro, Silvia Schoenau de Azevedo, Marcelo Jenné Mimica, Rafaela Fabri Rodrigues, Mauricio Magalhães, Bruno Fernandes dos Anjos, Gabriel Fernando Todeschi Variane

**Affiliations:** ^1^ Protecting Brains & Saving Futures, Clinical Research Department, São Paulo, Brazil; ^2^ Division of Neonatology, Department of Pediatrics, Irmandade da Santa Casa de Misericórdia de São Paulo, São Paulo, Brazil; ^3^ Santa Casa de São Paulo School of Medicine, São Paulo, Brazil

**Keywords:** mixed reality (MR), augmented reality (AR), immersive technologies, neonatology, neonatal care, newborns and infants, sarnat score

## Abstract

Mixed reality (MR) and augmented reality (AR) technologies bridge elements of the real and virtual worlds, emerging as tools that allow users to engage with digital cues to aid with tasks encountered in the physical environment. Thus, these holographic-based innovations are potential tools to support real-time patient care. The applications of MR and AR in neonatal care remain significantly underexplored. In the present article, we highlight the applications of MR and AR across medical procedures, physical examinations, medical diagnoses, and telemedicine, further underscoring their transformative potential within neonatal care. The use of MR and AR can be relevant across diverse economic and clinical landscapes, and in-depth research is required to evaluate the advantages of these tools in caring for neonates.

## Introduction

Immersive technology approaches are becoming more prominent in the healthcare field. Virtual Reality (VR), a widely known innovation, allows individuals to immerse themselves in a completely simulated environment (1). These computer-generated settings enable unique educational opportunities that simulate scenarios in surgical and clinical care (2, 3). However, VR completely excludes the real-world environment, making its application challenging in tangible, live medical care. For that reason, in this article we focus on the emerging approaches of Augmented Reality (AR) and Mixed Reality (MR).

In contrast to VR, AR allows the projection of digital content in real environments, expanding the visual reality with virtual cues (4). AR has since been further developed into a mixed reality (MR) technology, enabling virtual content to interact with objects in the physical world (5, 6). The holographic-based technologies of AR and MR allow users to interact with the virtual elements to assist with tasks encountered in the physical environment. Thus, the overlap of AR and MR (AR/MR) enables their application to live, real-world patient care.

Despite being widely explored, especially among simulated surgical procedures, the implications of applying AR/MR approaches to the care of neonatal patients necessitate more research (7, 8). The field of neonatology faces many challenges, such as heterogeneity of care and a lack of skilled interventions (9–12). AR/MR have the potential to minimize these disparities by positively contributing to medical procedures, physical examinations, clinical diagnoses, and telemedicine. For instance, instant access to holographically presented protocol instructions, such as neurological exams, may further globalize medical expertise. Therefore, further attention is warranted to assess the advantages of AR/MR technology in neonatal care.

This position paper aims to explore the applications of AR/MR in the clinical care of patients, with a focus on highlighting the prospects of integrating such initiatives into neonatal care. Additionally, we share our experience applying a trial version of an MR-based solution, as a proof-of-concept experience, in the neonatal intensive care unit (NICU) of a tertiary hospital in Brazil.

## AR/MR for medical procedures

AR/MR devices have been broadly explored as a tool in surgical specialties, with orthopedic surgery and neurosurgery techniques being the most commonly explored with immersive technology (7). The holographic-based imaging of AR/MR paves the way for various applications within surgery and surgical training. For instance, AR/MR devices may augment traditional image-guided interventions such as laparoscopy (13).

Unlike VR systems, AR/MR could offer the ability to provide see-through visualization. AR/MR may superimpose a clinical image, such as previously obtained CT or MR imaging data, directly on the patient (14–16). Gibby and colleagues explored the application of such a tool for interventional spine procedures across 10 patients, with promising results of AR navigation within such a context (16). The see-through visualization ability of AR/MR could provide access to underlying anatomy without physical incisions and may guide surgeons during pre-operative planning and intra-operative navigation for minimally invasive procedures (17–20). For instance, Wang and colleagues have depicted the clinical potential of AR surgical visualization within a knee arthroplasty context in an experimental environment (19). Under a similar laboratory setting with 80 lumbar spinal vertebra models, Dennler et al. found that utilizing AR may improve the precision of surgical pedicle screw insertion (20).

Moreover, the ability to render a 3D medical image into an AR/MR device could provide useful depth perception and spatial awareness of vital anatomical structures that may not be obtained through a conventional 2D image (21–24). Even during open surgical procedures, AR/MR could aid with actions such as blood vessel searches (25). While still a novel concept and mainly explored among phantom and simulated scenarios, surgical approaches guided by AR/MR carry the potential to reduce incision sizes, diminish potential errors, and reduce operative duration.

Beyond invasive interventions, AR/MR can also contribute to non-surgical clinical procedures. During ultrasound-guided examinations, for instance, an AR/MR device can contribute to the spatial orientation during the procedure as it may display the ultrasound image at the exact site or proximal to the anatomic location of interest (26). Additionally, AR/MR may enhance other invasive procedures, such as intubation or catheter placement (27, 28). Beyond its see-through visualization ability, AR/MR could also provide out-loud or holographic-based instructions for the accurate completion of procedures, a feature particularly relevant for healthcare trainees (29, 30).

## AR/MR for physical examination and clinical diagnosis

AR/MR can also be utilized as a tool for clinical diagnosis and examinations at the patient's bedside. Utilizing an AR/MR device for a 3D visualization of a clinical image instead of a conventional 2D view is an emerging reality, as discussed in the previous section (21–23). Immersive technology-based 3D imaging may be beneficial for diagnoses at the patient's bedside. For instance, Venson and colleagues have previously highlighted the value of immersive technology in identifying fractures through a 3D volumetric image visualization (31). Additionally, AR/MR can offer the ability to interact with clinical images hands-free, allowing for a simultaneous examination of the patient and a more detailed image inspection (e.g., zoom-in) (22). As investigated by Butaslac et al. and Klinker et al., AR/MR tools may also provide insights into the physical exam that include the ability to trace angular trajectories of joints or measure the size of wounds (32, 33).

Further, AR/MR technology could contribute to how healthcare providers visualize, monitor, and interact with a patient's medical data. Tanbeer & Sykes have presented a holographic-based system that can allow the observation of trends in vital signs, access to multiple vital modalities simultaneously, digital access to predictive risk scores such as the Hamilton Early Warning Score (HEWS), and instant availability of blood work results (34). The ability to holographically visualize medical data upon visiting a patient paves the way for an uninterrupted overview of vital parameters and information while freeing the hands for needed interventions (35–37).

Holographic-based projections from an AR/MR device have also been applied for educational and guidance purposes during physical exams. Concurrent with a physical examination, the clinician may have access to holographically presented reference videos, written instructions, and pathological images as an aid during care (38, 39). This could prove extremely relevant as the literature emphasizes that multimodal learning, such as an AR/MR approach, is more effective than conventional didactic methods (40, 41).

## AR/MR and telemedicine

Immersive technology has also been explored as a telemedicine tool (42, 43). Current AR/MR devices present video-conference capabilities, allowing a remote individual to communicate and access a real-time transmission of the AR/MR user's viewpoint (44). This communication medium enhances the remote individual's sense of presence, allowing the AR/MR device user to relay information without looking away from a context or maneuvering a device (45).

Teleconsulting via AR/MR has previously been applied during medical training and in the intensive care unit (ICU) environment, revolutionizing how medical experts provide consultations and assist trainees (44, 46, 47). This proves even more applicable to rural or disadvantaged settings with limited medical expertise. As a proof-of-concept study, Sirilak & Muneesawang developed a system based on holographic and AR systems to provide consults to a typical ICU. The findings from surveys across 17 medical consults demonstrated the reliability and efficiency of AR/MR systems for remote consultations in such a context (46). Meanwhile, Mistuno et al. assessed remote surgical mentoring utilizing MR through four simulated procedures performed on a craniofacial model, with qualitative feedback indicating various advantages and improvable aspects. Notably, the capability of teleguidance by projecting lines or arrows into the operator's field of view was a key factor for effective surgical telementoring (44). Aiding medical settings via AR/MR has further been proposed across other scenarios, such as telementoring for central venous catheter placement or remote emergency assistance (48).

By observing the first-person viewpoint of the AR/MR user, a remote medical expert may also have more practical and instant access to a specific patient's vitals and medical records (46). The application of AR/MR devices as a telemedicine tool could reduce variability in medical practice and improve patient outcomes, especially in healthcare settings with a shortage of specialized care (49). Furthermore, such an approach could also potentially contribute to cost-effectiveness (50), along with limiting the exposure of vulnerable patients to additional staff that can otherwise accompany cases remotely.

## Prospects of applying AR/MR tools to neonatal care

AR/MR technologies present various clinical applications, as depicted in Table 1. Yet, such applications to the care of neonates remain largely unexplored (7). AR/MR presents the potential to tackle the multifactorial challenges that impact neonatal care. Research estimates that simple and effective interventions could prevent most of the approximately 2.5 million neonatal deaths yearly, and many neonates who survive are at risk of developing neurological impairments due to the lack of well-known interventions (51). While low and middle-income countries present the highest rates of neonatal mortality and abnormal neurological outcomes, evidence also sheds light on the high variability of neonatal care across hospitals in high-income countries (9–11, 52). Overall, there is an urgency to improve and standardize neonatal care globally.

**Table 1:** Augmented and mixed reality studies referenced.

Citation	Study summary	Study population	Analyzed parameters	Outcomes	Conclusions
Tanbeer & Sykes (34)	An MR system (*MiVitals*) that integrates real-time vital signs, generates holographic visualizations, and allows healthcare professionals to interact with medical information holographically was presented.	A total of 37 participants evaluated the system. Twelve active nursing students, one practicing medical doctor, one medical software developer, four computer science professors, and other students.	The holographic application was evaluated in two distinct ways: 1. A qualitative survey design; and 2. The System Usability Scale.	The System Usability Scale study yielded a score of 84, indicating high usability. Participants reported comfort in using the system.	At the time of publication, authors stated that MiVitals stood out as the only system offering comprehensive MR visualizations for all vital signs, physiological signals, and an early warning score for patient monitoring.
Fei et al. (28)	An AR system that allows the overlay of intubation tools and internal airways, providing real-time guidance during the procedure, was presented.	A child manikin was used to develop and test the system.	Evaluation of proposed system.	Three-dimensional CT images were acquired from the manikin. Different tissues were segmented to generate the 3D models that were imported into a program to build the holograms.	The AR-guided system has potential applications in tracheal intubation guidance.
Orchard et al. (47)	MR as a clinical communication tool within a simulated on-call setting was evaluated.	Thirty medical trainees communicated with a remotely located consultant.	The quality of communication (MR vs. traditional telephone) was evaluated objectively and subjectively.	Clinical communication utilizing MR scored statistically higher than telephone. Qualitative feedback highlighted ease in use and the added value of MR.	Utilizing MR for clinical communication may be superior to conventional communication methods.
Tsang et al. (36)	Assessed adherence to guideline utilizing an AR electronic decision support tool during standardized simulated neonatal resuscitation.	A total of 18 professionals responsible for neonatal resuscitation were randomized to the intervention group and 11 to the control group.	A standardized neonatal resuscitation scenario was performed and assessed for adherence to the Newborn Life Support (NLS) algorithm. Algorithm deviation and time to the execution or completion of critical steps were also evaluated.	Errors of commission were committed less frequently with the electronic decision support tool. Time to initiation or completion of key steps showed no significant differences between groups.	An electronic decision support tool has the potential to improve guideline adherence for healthcare professionals.
Rebol et al. (48)	A MR real-time communication system for ultrasound-guided placement training of a central venous catheter was studied.	Five instructors and 20 learners. Training was performed on a phantom.	The study compared the MR system against training with video communication software. The results were analyzed using surveys and video recordings.	Feedback from instructors and learners about the MR system was very positive. Video and MR training resulted in equal workload.	MR offers new possibilities for visual communication that extend beyond conventional video teleconference-based training.
Nguyen et al. (26)	AR as a display platform for ultrasound was evaluated.	Novices (engineers, medical students; *n* = 8) and experts (residents, fellows, and practicing physicians; *n* = 8) applied the application in a custom phantom.	A user study was performed to assess the effectiveness of the display platform. Tasks involving targeting simulated vessels using a needle were timed.	With AR visualization, the novices’ times improved by 17% but the experts’ times decreased by 5%. Results found that the application can enhance user experience and simplify hand–eye coordination.	An AR application has the potential to improve efficiency and effectiveness of ultrasound-guided examinations and procedures.
Lau et al. (21)	The value of MR and 3D printing in aiding congenital heart disease diagnosis, education, and procedure was evaluated.	Two cardiac tomography angiography scans (atrial septal defect and double outlet right ventricle) were converted into 3D-printed heart models and MR models. Thirty-four cardiac specialists and physicians provided evaluations.	Specialists filled out a questionnaire to rank each modality.	MR models were ranked as the best modality and were significantly better than DICOM images for diagnosis, depth perception, spatial relationship, pathological visualization, and pre-operative planning.	MR and 3D printing models are superior to conventional DICOM images in the visualization and management of congenital heart disease.
Kitagawa et al. (13)	A navigation system with 3D models generated by MR was evaluated as an intraoperative tool during laparoscopic cholecystectomy.	The MR device was utilized for 9 patients; conventional two-dimensional images were utilized for 18 patients.	Surgical outcomes such as operative time, blood loss, and perioperative complication rate were measured, and a questionnaire was used for subjective evaluation.	Median operative time was longer when utilizing MR. No intraoperative blood loss or perioperative complications occurred.	This study highlights that 3D MR models are a feasible tool during laparoscopic cholecystectomy.
Velazco-Garcia et al. (37)	A framework for interactive immersion with data, integration of image processing and analytics, and rendering and fusion with a holographic AR system is described.	Five clinical personnel contributed to setting the technical specifications of the platform for three clinical paradigms: brain, prostate, and cardiac. Subjective assessment of ergonomics and functionalities were completed by 14 medical personnel and 18 engineers/scientists for minor improvements.	Factors such as the speed of image change, total time for image reconstruction, user experience, and battery consumption were tracked.	Performance shows that the system can maintain one image per second with a resolution of 512 × 512. The system can also modify visual properties of the holograms at 1 update per 16 milliseconds (62.5 Hz) while providing enough resources for the segmentation and surface reconstruction tasks without hindering the head-mounted displays.	A framework without major technical challenges is proposed and has the potential to be used as a foundation for future AR medical systems.
Shah et al. (35)	A MR system that provides automatic patient data retrieval, interactive data visualization, and mid-air data entry mechanism was presented.	Functional testing was performed by 3 non-healthcare professionals based on dummy patient profiles (*n* = 4).	Functional testing was done to qualitatively evaluate the potential of the system.	The system was determined to be useful for patient data management.	An MR system has the potential to free up time that is now used for data management.
Sanchez-Margallo et al. (38)	The study presents two solutions based on MR holographic technology that aims to improve training and planning in minimally invasive surgery.	Six experienced surgeons in urology evaluated the proposed training solution. Two experienced laparoscopic surgeons evaluated the proposed surgical planning solution. The surgical planning solution was used during laparoscopic renal tumorectomy in an experimental model and video-assisted right upper lobectomy in an adult patient.	Participants completed a questionnaire to evaluate its use.	The solution for medical training was considered useful for training in urological anatomy. The solution developed for surgical planning facilitated the surgical approach.	A MR-based tool can assist with clinical training and real-time procedure.
Gehrsitz et al. (23)	Assessed a cinematic, MR system in the preoperative planning of cardiac surgery.	Twenty-six patients underwent cross-sectional imaging in preparation for surgery.	A 2D image, a 3D-printed model, and a cinematic rendering (CR) hologram were presented to the surgeon of the case. The surgeons completed a questionnaire for each technique.	CR-holograms surpassed 2D-monitor imaging in all categories. CR-holograms were superior to 3D prints in all categories, especially for in-depth perception and the representation of the pathology. 3D imaging reduced the intraoperative preparation time.	Holographic visualization of data via mixed-reality technology could allow a previously unattained level of comprehension of anatomy and pathology in preoperative planning.
Buch et al. (18)	A pipeline to construct, visualize, and register intraoperative holographic data of patients during spinal fusion surgery was presented.	Applied across 13 patients who underwent spine fusion surgery.	In phase 1, the pipeline was developed. In phase 2, registration accuracy methods were assessed.	Custom controller-based registration significantly reduced the mean registration error compared to the native hand-gesture registration. Accuracy improved over time (*p* < .0001).	Holographic MR has the potential to revamp intraoperative visualization, however, improvements are needed for clinical utility.
Schoeb et al. (39)	Evaluated a MR guidance system for instructing a practical medical procedure.	A total of 164 medical students utilized the tool.	In a randomized, single-blinded prospective trial (instructor vs. MR), the study assessed learning outcome via a standardized OSCE (objective structured clinical examination). A qualitative questionnaire was also filled out.	Results reveal similar outcomes in the self-evaluation and a slightly better learning outcome in the MR group in the OSCE exam. The control group gave higher ratings when evaluating the quality of instructions.	MR is a promising tool for instructing practical skills and has the potential to yield superior learning outcomes.
Klinker et al. (33)	The application of a smart glass tool during wound treatment was evaluated.	A total of 45 health care workers with wound management experience.	User performance, satisfaction, and technology acceptance in the context of wound documentation were assessed.	Smart glass-based documentation systems are viewed significantly more favorable in terms of Performance Expectancy, Behavioral Intention, and Satisfaction compared to existing documentation processes.	AR has the potential to significantly innovate the health care sector across treatments.
Katayama et al. (25)	The projection of blood vessels onto the surgical field using AR was assessed.	A 13-year-old adolescent girl with macrodactyly of the right foot and a 67-year-old woman with malignant peripheral nerve sheath tumor in the right forearm.	Two types of projection for holograms were evaluated: type 1—where the body contours were projected as a line, and type 2—where the body surface was projected as meshed skin type.	Projection type 2 provided a better understanding of the 3D anatomic findings and deformation characteristics.	The 3D visualization of blood vessels from the body surface is feasible utilizing AR.
Gibby et al. (16)	Evaluated whether superimposing imaging data on a patient positively impacts minimally invasive spine procedures.	A total of 18 procedures were performed across 10 patients undergoing an interventional spine procedure. Comparative controls were generated using a phantom model (*n* = 32).	Target error, distance to the target, and target size were measured for both phantom and clinical groups.	Target error showed no significant difference between groups. The distance to the target site and the target size had no effect on target acquisition.	AR navigation is an accurate and valuable method for planning within a percutaneous image-guided spinal procedure context.
Dennler et al. (20)	The applicability of AR in pedicle screw insertion was assessed in a laboratory setting.	Eighty identical models of a third lumbar spinal vertebra were drilled by two spine surgeons and two novice surgeons.	Half of the pedicles were drilled using the free-hand method and half using the AR method. Precision was evaluated.	The AR method improved the precision of drilling pilot holes for pedicle screws and decreased the impact of surgeon's experience.	The application of AR within the accuracy of pedicle screw insertion is promising and its clinical benefit should be investigated.
Butaslac et al. (32)	AR as an *in situ* visualization data support tool in rehabilitation was evaluated.	Rehabilitation therapists (number unspecified) evaluated the tool.	Qualitative user research validated the effectiveness and feasibility of the proposed system.	The feedback from the therapists was positive.	Immersive technology has the potential to be applied in everyday clinical rehabilitation and training.
Azimi et al. (27)	MR, as a system that overlays a target directly on the patient's anatomy, was evaluated within a catheter insertion context.	Ten participants with engineering or medical backgrounds applied the studied tool on a phantom.	Accuracy and precision of the insertions as well as the usability of the system were evaluated.	The MR-based navigation system presented high perceived usability and improved targeting accuracy by more than 35%.	MR has a high potential for improving bedside surgical procedures.
Wang et al. (19)	An AR approach for image guidance was proposed.	A virtual femur and a virtual tibia from CT data were overlaid in the view of HoloLens along with a virtual surgical guide model.	Distances between the true osteotomy planes and the position of the overlaid virtual surgical guides were evaluated for accuracy.	The overlay errors for the femur and tibia were both about 2.5 mm, which meets the clinical surgical requirement.	AR has the potential to provide minimally invasive total knee arthroplasties with intuitive surgical visualization.
Mitsuno et al. (44)	The use of telementoring via Skype and MR in craniofacial surgery was evaluated. Additionally, 3-layer facial models were presented.	One resident performed simulated surgery while mentored by a more experienced surgeon.	Voice communication speed as well as qualitative surgical reference data were evaluated.	There was no delay in voice communication and a minimal delay in the video. The resident was able to confirm the main landmarks of the surgical field. The mentor could send appropriate instructions by voice, could point out a specific part, and could draw lines on the 2-dimentional images pasted on the operator's field of vision.	A telementoring system could positively influence mentorship in the field of craniofacial surgery.
Brun et al. (22)	Evaluated the preoperative diagnostic use of MR holograms of individual 3D heart models from standard cardiac computed tomography angiograms images.	Assessed with a patient with double outlet right ventricle and transposition of the great arteries. The model was evaluated by 36 pediatric heart team members.	A diagnostic and quality rating questionnaire was filled out. Morphological and diagnostic output from the hologram was assessed. App tools such as hologram rotation, scaling, and cutting were rated.	All but one correctly identified the diagnosis and all but two correctly identified the transposition of the great arteries. Anatomy identification, diagnostic output, and experience rating were high.	MR may have a high diagnostic value for congenital heart disease and could contribute to the understanding of complex cases.
Alismail et al. (30)	Evaluated AR glasses as a tool in performing a simulated intubation procedure.	A total of 32 subjects participated in this study (Respiratory, *n* = 19; Medical Doctor, *n* = 11; Sleep Technology, *n* = 2),	Guideline adherence and time to ventilate was assessed.	The AR group took longer to ventilate than the non-AR group. There was a higher percent guideline adherence for the AR group.	AR is promising in improving endotracheal intubation.
Aaskov et al. (14)	A technology that views a participant's own x-ray superimposed on their back was evaluated.	A total of 13 participants with a pre-existing anteroposterior lumbar x-ray.	Utilizing the superimposed image, vertebral levels were identified and validated against spinous process locations acquired by ultrasound. The process was repeated 1–5 days later.	The system provided on-target projections with respect to vertebral levels 73% of the time with no significant difference between testing sessions. The average repeatability for all vertebral levels between testing sessions was 77%.	A technology that projects x-rays directly onto the skin is feasible for identifying underlying anatomy.
Sirilak & Muneesawang et al. (46)	An E-consultation system leveraged on holographic and AR systems was evaluated for intensive care unit (ICU) consults.	Included 7 cases of Cardiovascular thoracic Surgery, 4 surgery cases, 3 emergency trauma cases, and 3 cases in the ICU unit.	Satisfaction and usability surveys evaluated the system.	Out of a maximum score of five, the average satisfaction was 4.37 for system usability, 4.5 for the benefits identified, and 3.87 for the average satisfaction.	The present study can justify the use of immersive technology as E-consulting systems in ICU centers.
Huang et al. (29)	AR glasses for central line simulation were evaluated.	A total of 32 adult novice central line operators. Medical doctors, respiratory therapists, and sleep technicians were recruited from the medical field.	Subjects were randomized to either simulation using the AR glasses or simulation using conventional instruction. Adherence level and mean procedure time were evaluated.	There was a significant difference in the adherence level between the two groups favoring the AR group (*p* = 0.003). There was no significant difference between the groups in procedure time.	AR is a promising educational tool in central venous catheter placement.
Agten et al. (17)	Assessed AR–guided lumbar facet joint injections.	A spine phantom was built. Two radiologists independently performed 20 AR-guided and 20 CT-guided facet joint injections each.	Accuracy and time to place needles were compared between groups.	In total, 39/40 of AR–guided needle placements were either perfect or acceptable compared with 40/40 CT-guided needle placements (*p* = 0.5). Time to final needle placement was substantially faster with AR guidance.	AR–guided facet joint injections are safe, feasible, and accurate in an experimental scenario.
Wang et al. (45)	Assessed the use of AR in capturing the first-person view of a simulated rural emergency room.	Twelve participants with minimal Point of Care Ultrasound (PoCUS) experience enrolled in the study with a HoloLens setup. Other twelve participants were assigned to complete the remote PoCUS training study using a “full telemedicine setup”.	The study explores the utility of the system from the trainees, mentor, and objective observers’ perspectives.	The AR setup and Leap Motion did not show significant statistical difference when compared to a full telemedicine setup.	An AR telemedicine platform yields further evaluation based on the insights of this study.
Mojica et al. (24)	A holographic 3D visualization of MRI data was presented for planning neurosurgical procedures.	Three clinical personnel, one neurosurgeon, and six computer science and engineering school faculty and graduate students tested the system. One healthy volunteer and one meningioma patient were the cases studied.	Qualitative evaluation of users and quantitative evaluation of HoloLens processing capability (e.g., CPU usage) were performed.	The processing of textures for the Sagittal plane took the longest. A holographic visualization of 3D MRI data offers an intuitive and interactive perspective of the brain, subjectively found to be superior to desktop-based imaging.	Immersive technology may be an unparalleled tool for preoperative planning of neurosurgeries.

AR, augmented reality; MR, mixed reality; 3D, three-dimensional; MRI, magnetic resonance imaging; CT, computed tomography.

AR/MR's ability to holographically provide instructions, reference media, and digital access to diagnostic assessments while the user remains hands-free for clinical examinations may further improve and homogenize neonatal care. The benefits could be even more significant for managing neonatal emergencies in settings that lack the required expertise for the appropriate response. The application of digital clinical support tools has already shown the potential to improve patient care in different medical scenarios (30, 53, 54). Particularly in neonatology, digital tools have been applied across simulated resuscitation and intubation training, with findings highlighting improvement in guideline adherence (36, 55, 56). Thus, the application of AR/MR should be further explored and invested across various protocols in tangible neonatal care with existing patients.

Beyond the holographic projection of protocol information, the possibility of an AR/MR-based video communication also proves relevant. Access to a real-time transmission of the AR/MR user's viewpoint could redesign how medical expertise reaches underprivileged medical settings globally. Even for on-site consults and case discussions, an AR/MR videoconference could limit the exposure of vulnerable infants to infections and provide a better viewpoint to trainees who would otherwise have limited visualization due to a crowded room. Conversely, trainees could perform examinations being remotely monitored by experts.

**Figure 1:**
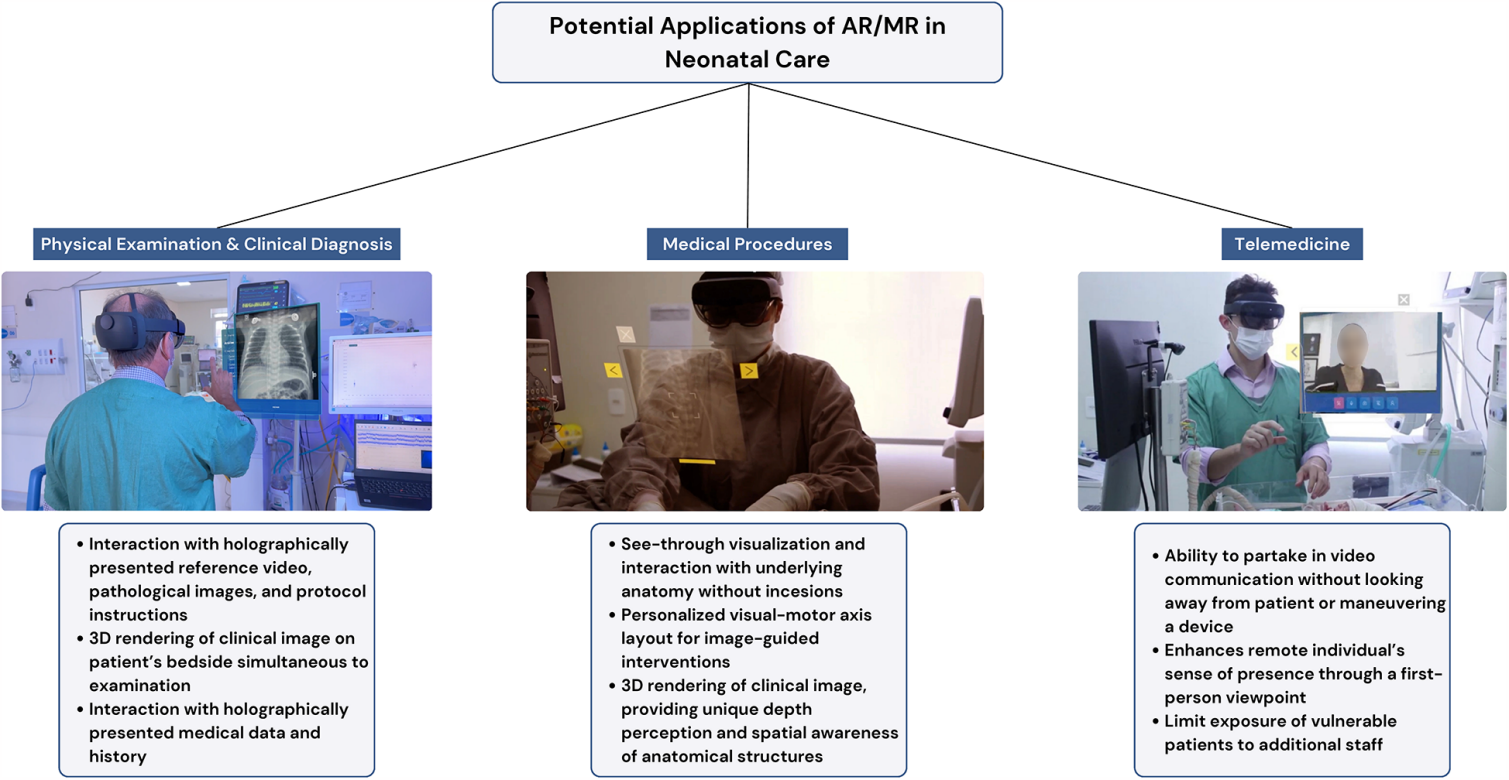
Potential applications of augmented and mixed reality (AR/MR) in neonatal care.

Furthermore, utilizing AR/MR-based guidance during medical procedures performed in neonatal patients is another area that yields further exploration. The smaller anatomy, fragile tissues, and low blood volume that neonates present require further specificity upon performing medical procedures. The possibility of AR/MR-based software to superimpose a clinical image onto the patient and allow a see-through visualization of underlying anatomy without incisions could prove particularly relevant when dealing with the immature anatomy and physiology of newborns. Additionally, rendering a 3D medical image into an AR/MR device could provide a level of comprehension and visualization previously unreached, contributing to preoperative planning (23). The added challenge of managing pediatric anatomy underscores the need for additional initiatives that explore AR/MR-guided medical procedures (57, 58). Overall, AR/MR technology may address the multifactorial challenges impacting neonatal care, as illustrated in Figure 1.

## A practical example of implementing an MR-based solution in the NICU

To improve and further homogenize the care of high-risk neonates, our group developed an MR-based software entitled *NeonLens*, an immersive technology solution to assist healthcare professionals in the NICU. NeonLens (Protecting Brains & Saving Futures, São Paulo, Brazil) is compatible with MR devices and provides specialists with assisted guided support through clinical protocols, along with enabling remote hands-free video communication with specialists to improve neonatal care. As part of a preliminary proof-of-concept experience, we applied the NeonLens across the following scenarios in a Brazilian NICU:

○ Medical Sign-out Rounds and Clinical Case Discussions:
  Medical sign-out rounds at our institution can be highly crowded with physicians, neonatal fellows, pediatric residents, interns, nurses, and other healthcare professionals. Due to this reality, trainees and healthcare providers may encounter an affected view of the patient being examined in the NICU, directly impacting case discussions during rounds. Thus, NeonLens has been proposed as a solution within this context. A neonatal fellow at the bedside, wearing Microsoft HoloLens 2 with NeonLens, conducted sign-out rounds and clinical case discussions while being assisted in real-time by a senior physician located remotely. The NeonLens user had a holographic video and audio of the remote medical expert while examining the patient and the surrounding monitors. Meanwhile, the remote senior physician had access to the user's viewpoint and audio through a video call on a computer. Other trainees and physicians were also able to accompany the discussion both locally or remotely.
○ Clinical Decision Support Tool for the Modified Sarnat Exam:
  The Modified Sarnat exam is a common assessment tool used to evaluate the severity of hypoxic-ischemic encephalopathy in neonates, and previous literature has reported high heterogeneity among professionals when performing the exam (59). Our group envisioned using NeonLens as an opportunity to reduce this variability and enhance the accuracy of the neurological exam. The physicians experienced using Microsoft HoloLens 2 with NeonLens to access detailed holographic, multimedia-guided instructions for performing the Modified Sarnat exam. The device provided the diagnosis of the Modified Sarnat exam based on the user's response to each exam item. Simultaneously, the NeonLens user at the infant's bedside had remote expert guidance *via* holographic video and audio to guide the user through each step of the clinical exam.

During this proof-of-concept experience, nine neonatology fellows received training and utilized the NeonLens solution across 45 medical sign-out rounds and 12 Modified Sarnat exams, totaling 34 h of use. As this was a preliminary proof-of-concept implementation, user experience was recorded qualitatively. Overall, the positive aspects were the reassurance of having a remote specialist and the intuitive, user-friendly interface that provided instant access to information that guided the physical exam. The challenges included difficulty hearing other healthcare professionals while using the tool and uncertainty about the exact remote expert view of the newborn. Repeated use led to greater confidence and ease.

## Discussion

AR/MR technology is a tool with the potential to revolutionize medicine. As explored in this manuscript, such immersive technology approaches present several applications within a medical context, from the 3D rendering of clinical images to holographically provided protocol information (21, 35). The widespread applications of AR/MR can significantly contribute to clinical training and current advancement warrant its application within tangible, real-world patient care scenarios (56). Holographic access to protocol information, reference media, and video communication with a remote expert—all while remaining hands-free to perform a clinical exam or procedure—could transform and homogenize medical care.

AR/MR should be explored as a strategy to tackle global challenges in neonatal care. While underdeveloped countries present higher rates of newborn mortality that may be due to the lack of basic resources, high-income nations also present challenges, such as the high variability of care among settings (9–12, 52). Therefore, as previously explored, the applicability of AR/MR as a tool to improve care can be envisioned across diverse contexts. For instance, AR/MR technology may expand the mentorship of more experienced physicians to less specialized care settings. Further, presenting protocol instructions through an AR/MR device could ensure that expert physicians across hospitals adhere rigorously to guidelines. Ultimately, the transformative potential of AR/MR may contribute to the reduction of neonatal mortality and morbidity. Future studies should investigate the impact of implementing AR/MR strategies to improve neonatal outcomes.

Understanding the current challenges of AR/MR initiatives in medicine is essential for developing a plan and implementing its use. Current AR/MR software devices present a battery life and field of view that may not be optimal for specific clinical scenarios, especially for more extended examinations and procedures (7). While such limitations are currently being addressed, healthcare professionals also report concerns about the cost associated with immersive technology (8, 60). Thus, the cost-effectiveness of AR/MR also needs to be further evaluated. For example, AR/MR's potential to homogenize medical care may potentially reduce the length of hospitalization and the number of medical tests required, warranting economic analyses of cost-minimization and cost-consequence of its application. Bergin & Craven have recently published the first review addressing the cost-effectiveness of extended reality technologies (an umbrella term including virtual, augmented, and mixed reality), which addressed that such innovations are promising in a value-based healthcare context (61). These findings are encouraging, and future investigations should specifically review the value of AR/MR within various contexts.

Another barrier to AR/MR technology is the limitation in rendering highly detailed clinical imaging data and superimposing such images for pre-operative planning and procedural guidance. Present literature cautions against using this tool for procedures requiring sub-millimeter accuracy as it may be necessary when caring for neonates (7). However, its accuracy still proves relevant for less critical scenarios such as anatomy and surgical training (62, 63). Additional investigations also highlight issues with depth perception upon a 3D visualization and note that cybersickness may be linked to prolonged use (7, 8, 64, 65). While these limitations indicate areas that future work should explore, our preliminary implementation of MR in the NICU did not present significant obstacles.

Nevertheless, the mentioned challenges do not diminish the unparalleled potential of AR/MR technologies. While the surgical applications of AR/MR may necessitate further research, other aspects of the technology can be considered ready for deployment, especially in settings with an urgent need for enhanced medical care. The holographic projection of protocol information and reference media, along with the one-of-a-kind video-communication tool, as we explored in our preliminary implementation of the NeonLens, are currently highly applicable to tangible neonatal care. Improving newborn care can have profound individual and systemic benefits, and key aspects of AR/MR technology are ready to contribute to this mission.

To conclude, AR/MR technology presents the potential to positively impact several facets of medical care, including medical procedures, physical examination and diagnosis, and telemedicine. Each component warrants individual evaluation—while some features may be considered ready for integration, others yield further research. The applications of AR/MR in neonatal care should be regarded as relevant across diverse economic and clinical landscapes. Future investigations should assess each component of this technology in various medical settings, along with integrating AR/MR with other rising tools like artificial intelligence. Furthermore, emerging projects should explore the cost-effectiveness and impact on short- and long-term clinical outcomes.

## Data Availability

The raw data supporting the conclusions of this article will be made available by the authors, without undue reservation.
